# Supplementary material for: Analysis of PPARγ Signaling Activity in Psoriasis
Source: Int J Mol Sci. 2021 Aug 10;22(16):8603. doi: 10.3390/ijms22168603 (PMC8395241; doi:10.3390/ijms22168603)
Supplement: Supplementary file 1 [file ijms-22-08603-s001.zip › Supplemental materials_Analysis of PPARg signaling activity in psoriasis/Pathway models/Models images and html files/Model of PPARG related pathways in psoriasis (short version)/26415.html]

IL36A


# Protein IL36A

|  |  |
| --- | --- |
| URN | urn:agi-llid:27179 |
| Total Entities | 0 |
| Connectivity | 286 |
| Name | IL36A |
| Description | interleukin 36 alpha |
| Notes | The protein encoded by this gene is a cytokine that can activate NF-kappa-B and MAPK signaling pathways to generate an inflammatory response. The encoded protein functions primarily in skin and demonstrates increased expression in psoriasis. In addition, decreased expression of this gene has been linked to a poor prognosis in both hepatocellular carcinoma and colorectal cancer patients. [provided by RefSeq, Nov 2015] |
| Primary Cell Localization | Extracellular |
| Class | Ligand |

---

|  |  |
| --- | --- |
| Pathway | Proteins Involved in Psoriatic Arthritis |
|  | IL36 Receptor Antagonist (IL1F5) Deficiency (DITRA) |
|  | NOTCH -> Expression Targets in Skin |
|  | prarg negative regulators, ps-positive |
|  | PPARG negative regulators |
|  | pparg expr target\_regulators |
|  | Neighbors of disease exacerbation |
|  | Neighbors of tumor progression |
|  | Neighbors of lethality |
|  | Neighbors of neutrophil count |
|  | Neighbors of mortality rate |
|  | Neighbors of skin thickness |
|  | Neighbors of disease exacerbation |
|  | Neighbors of tumor progression |
|  | Neighbors of lethality |
|  | Neighbors of neutrophil count |
|  | Neighbors of mortality rate |
|  | Neighbors of skin thickness |
|  | Neighbors of cell development |
|  | Neighbors of immune response |
|  | Neighbors of inflammatory response |
|  | Neighbors of innate immune response |
|  | Neighbors of macrophage activation |
|  | Neighbors of adaptive immune response |
|  | Neighbors of chemotaxis |
|  | Neighbors of T-cell proliferation |
|  | Neighbors of cell adhesion |
|  | Neighbors of cell count |
|  | Neighbors of adipogenesis |
|  | Neighbors of cell motility |
|  | Neighbors of adipocyte differentiation |
|  | Neighbors of neutrophil recruitment |
|  | Neighbors of phagocytosis |
|  | Neighbors of cell growth |
|  | Neighbors of cell differentiation |
|  | Neighbors of psoriasis |
|  | Neighbors of rheumatoid arthritis |
|  | Neighbors of atherosclerosis |
|  | Neighbors of multiple sclerosis |
|  | Neighbors of inflammation |
|  | Neighbors of pneumonia |
|  | Neighbors of osteoarthritis |
|  | Neighbors of reperfusion injury |
|  | Neighbors of infection |
|  | Neighbors of macrophage infiltration |
|  | Neighbors of sepsis |
|  | Neighbors of dermatitis |
|  | Neighbors of colorectal cancer |
|  | Neighbors of systemic lupus erythematosus |
|  | Neighbors of interstitial fibrosis |
|  | Neighbors of neoplasm |
|  | Neighbors of epithelial cell |
|  | Neighbors of keratinocyte |
|  | Neighbors of macrophage |
|  | Neighbors of airway epithelial cell |
|  | Neighbors of pneumocyte |
|  | Neighbors of intestine epithelium cell |
|  | Neighbors of dendritic cell |
|  | Neighbors of neutrophil |
|  | Neighbors of bone |
|  | Neighbors of NF-kB family |
|  | Neighbors of interleukin |
|  | Neighbors of Notch |
|  | Model of PPARG signaling in psoriasis |
|  | PPARG negative regulators and targets |
|  | Model of PPARG related pathways in psoriasis (short version) |

---

|  |  |
| --- | --- |
| MedScan ID | 27179 |

---

|  |  |
| --- | --- |
| LocusLink ID | 27179 |
|  | 54448 |
|  | 296541 |

---

|  |  |
| --- | --- |
| Alias | FIL1E |
|  | MGC151479 |
|  | MGC129552 |
|  | interleukin 1 family, member 6 (epsilon) |
|  | interleukin IL1F6 |
|  | interleukin 1 family, member 6 |
|  | MGC129553 |
|  | interleukin 36, alpha |
|  | LOC83004 |
|  | RP23-176J12.4 |
|  | IL1F6 |
|  | MGC151481 |
|  | Il36a |
|  | interleukin IL-1f6 (fil-1-epsilon) |
|  | IL-1f6 (fil-1-epsilon) |
|  | OTTMUSP00000012798 |
|  | interleukin 1 family, member VI |
|  | FIL1(EPSILON) |
|  | FIL1 |
|  | IL-1F6 |
|  | IL1(EPSILON) |
|  | FIL1 epsilon |
|  | IL-1 epsilon |
|  | interleukin-1 epsilon |
|  | interleukin-1 family member 6 |
|  | interleukin-36 alpha |
|  | Il1f9 |
|  | IL-1H1 |
|  | IL1RP2 |
|  | Fil1epsilon |
|  | interleukin 1 family, member 9 |
|  | interleukin-1 homolog 1 |
|  | Il1h1 |

---

|  |  |
| --- | --- |
| GO ID | 0005125 |
|  | 0005149 |
|  | 0071222 |
|  | 0019221 |
|  | 0006955 |
|  | 0006954 |
|  | 0045087 |
|  | 0030593 |
|  | 0043123 |
|  | 0046330 |
|  | 0032755 |
|  | 0005576 |
|  | 0005615 |
|  | 0002376 |
|  | 0001819 |
|  | 0010469 |

---

|  |  |
| --- | --- |
| KEGG ID | hsa:27179 |
|  | mmu:54448 |

---

|  |  |
| --- | --- |
| Organism | Homo sapiens {Organism urn:agi-taxid:9606} |
|  | Mus musculus {Organism urn:agi-taxid:10090} |
|  | Rattus norvegicus {Organism urn:agi-taxid:10116} |

---

|  |  |
| --- | --- |
| Mouse chromosome position | 2 16.26 cM |

---

|  |  |
| --- | --- |
| OMIM ID | 605509 |

---

|  |  |
| --- | --- |
| Rat chromosome position | 3p13 |

---

|  |  |
| --- | --- |
| Hugo ID | 15562 |

---

|  |  |
| --- | --- |
| Human chromosome position | 2q14.1 |

---

|  |  |
| --- | --- |
| Swiss-Prot Accession | Q9UHA7 |
|  | Q9UHA7.1 |
|  | Q149U6 |
|  | Q9JLA2 |
|  | Q9JLA2.1 |
|  | D4A1I2 |
|  | B2RAD9 |
|  | Q53SR7 |
|  | Q5BLR4 |
|  | Q7RTZ8 |

---

|  |  |
| --- | --- |
| GenBank ID | NC\_000002 |
|  | NM\_014440 |
|  | NP\_055255 |
|  | XM\_011510965 |
|  | XP\_011509267 |
|  | XM\_005263639 |
|  | XP\_005263696 |
|  | XM\_017003806 |
|  | XP\_016859295 |
|  | AC016724 |
|  | AAY14988 |
|  | AY944686 |
|  | AAX20113 |
|  | BN000002 |
|  | CAD29875 |
|  | CH471217 |
|  | EAW73614 |
|  | AF201831 |
|  | AAF25211 |
|  | AK314149 |
|  | BAG36836 |
|  | BC107042 |
|  | AAI07043 |
|  | BC107043 |
|  | AAI07044 |
|  | Q9UHA7 |
|  | NC\_000068 |
|  | NM\_019450 |
|  | NP\_062323 |
|  | AL732430 |
|  | CH466542 |
|  | EDL08163 |
|  | AF200493 |
|  | AAF69249 |
|  | AF206697 |
|  | AAG35671 |
|  | AK004061 |
|  | BAB23147 |
|  | AK224928 |
|  | AK224929 |
|  | AK224931 |
|  | AK224932 |
|  | BC117102 |
|  | AAI17103 |
|  | BC117104 |
|  | AAI17105 |
|  | KM205447 |
|  | AKA62187 |
|  | KM205448 |
|  | AKA62188 |
|  | Q9JLA2 |
|  | NC\_005102 |
|  | XM\_008761581 |
|  | XP\_008759803 |
|  | NM\_001106554 |
|  | NP\_001100024 |
|  | AC\_000071 |
|  | AABR07051212 |
|  | AAHX01020410 |
|  | CH474001 |
|  | EDL93670 |

---

|  |  |
| --- | --- |
| Swiss-Prot ID | IL36A\_HUMAN |
|  | IL36A\_MOUSE |

---

|  |  |
| --- | --- |
| Cell Localization | Secreted |

---

|  |  |
| --- | --- |
| Ensembl ID | ENSG00000136694 |
|  | ENSP00000259211.6 |
|  | ENST00000259211.6 |
|  | ENSMUSG00000026984 |
|  | ENSMUSP00000028361.4 |
|  | ENSMUST00000028361.4 |
|  | ENSRNOG00000005722 |
|  | ENSRNOP00000007540.4 |
|  | ENSRNOT00000007540.5 |

---

|  |  |
| --- | --- |
| MGI ID | MGI:1859324 |

---

|  |  |
| --- | --- |
| RGD ID | 1306869 |

---
